# Optimising the ECMO treatment regimen increases the survival rate for adult patients with acute fulminant myocarditis: A single-centre retrospective cohort study

**DOI:** 10.3389/fmed.2023.1146570

**Published:** 2023-03-16

**Authors:** Liusheng Hou, Hongkai Liang, Shiyong Zeng, Jianwei Li, Zhou Chen, Xiaozu Liao, Shaozhong Liu, Mingxing Li, Binfei Li, Yong Yuan

**Affiliations:** ^1^Department of Critical Care Medicine, Zhongshan City People’s Hospital, Zhongshan, Guangdong, China; ^2^Department of Anesthesiology, Zhongshan City People’s Hospital, Zhongshan, Guangdong, China; ^3^Department of Ultrasound Medicine, Zhongshan City People’s Hospital, Zhongshan, Guangdong, China; ^4^Department of Cardiovascular Center, Zhongshan City People’s Hospital, Zhongshan, Guangdong, China

**Keywords:** extracorporeal membrane oxygenation, acute fulminant myocarditis, cardiogenic shock, cardiac arrest, survival rate

## Abstract

**Background:**

Applying Extracorporeal membrane oxygenation (ECMO) to patients with acute fulminant myocarditis (AFM) reduces their mortality. The survival rate is 55.6-71.9% for adult AFM patients, which is lower than that for paediatric patients (63-81%). In our centre, the survival rate of ECMO for adult patients with AFM was 66.7% from January 2003 to 2012. In January 2013, the therapeutic regimen was optimised, and then the survival rate increased to 89.1% by January 2022. This article analyses the reasons for the improved survival rate following the optimisation of treatment protocols.

**Methods:**

The data for adult patients with AFM who underwent ECMO for a poor response conventional treatment from January 2003 to January 2022 were reviewed. According to different treatment regimens, the AFM patients were divided into an old and a new regimen group. Univariate and multivariate logistic regression analyses were performed on the data before and after ECMO.

**Results:**

Fifty-five patients were enrolled in the age (31.2 ± 11.3), including 24 males. Forty-nine patients were weaned successfully from ECMO [duration: (4.1 ± 1.8) d], all of whom were discharged from the hospital, with a survival rate of 89.1%. Compared with the old regimen group, the new regimen group had a shorter duration of shock to ECMO, a lower proportion of patients receiving extracorporeal cardiopulmonary resuscitation (ECPR), a lower Vasoactive Inotropic Score (VIS), and lower levels of lactic acid, and high-sensitivity troponin T before ECMO (*p* < 0.05). Compared with the old regimen group, after ECMO, the new regimen group had lower ECMO flow, lower proportion of left ventricular dilation and lower limb ischemia injury, the duration of ECMO was shorter, and significantly improved the survival rate, the difference was statistically significant (*P* < 0.05). The duration of shock to ECMO and VIS before ECMO were independent risk factors for the survival rate (*p* < 0.05).

**Conclusion:**

Early ECMO initiation in adult AFM patients with a poor response to conventional therapy and low-flow ECMO to meet metabolic needs can reduce serious complications affecting the prognosis, may be associated with better outcomes.

## 1. Introduction

Acute fulminant myocarditis (AFM) refers to myocardial inflammatory lesions accompanied by hemodynamic instability. Despite a low incidence, AFM arises rapidly and progresses quickly to refractory cardiogenic shock, malignant arrhythmia, and even cardiac arrest (CA), causing high mortality (1, 2). AFM is a reversible myocardial disease with a good long-term prognosis as long as patients live through the crisis of their illness (1–3). Therefore, a variety of life support techniques should be adopted to save the lives of AFM patients where possible.

Veno arterial Extracorporeal membrane oxygenation (ECMO) provides cardiopulmonary function support, which can not only enable patients to live through a crisis smoothly but also allow referral of patients with severe myocardial injury and poor reversibility for ventricular assist device or heart transplantation, markedly lowering the mortality rate of AFM patients (3–6). To further increase the survival rate of ECMO for AFM, many scholars have devoted themselves to searching for risk factors for weaning or prognosis (7–9). Despite these efforts, reports (number of cases > 50) have revealed that the survival rate of ECMO for adult patients with AFM is only 55.6-71.9% (9–11), which is lower than that for paediatric patients with AFM (63-81%) (12). Therefore, strategies to increase the survival rate for adult patients require further study.

As the first medical centre to carry out ECMO in China, our centre has applied ECMO to the treatment of AFM since 2003. By December 2012, the survival rate of ECMO for adult patients with AFM had reached 66.7% (10/15). To further improve the survival rate, the treatment regimen was optimised in January 2013, with modifications such as in-advance ECMO initiation, prophylactic placement of a distal perfusion catheter, the use of intravenous immunoglobulin and low-flow ECMO support. By January 2022, the survival rate of ECMO for adult patients with AFM (39/40, 97.5%) was significantly increased compared with that before 2012 and is higher (49/55, 89.1%) than that in recent reports.

This article analyses the reasons for the improved survival rate following the optimisation of treatment protocols and discusses the following issues: the timing of ECMO interventions in adult AFM, the prevention and treatment of serious complications affecting the prognosis of adult AFM, such as CA and extracorporeal cardiopulmonary resuscitation (ECPR) before ECMO and left ventricular dilatation after ECMO, and the use of intravenous immunoglobulin (IVIG). This article provides experience in improving the success of ECMO in the salvage of adult AFM.

## 2. Study design and methods

### 2.1. Case collection and grouping

#### 2.1.1. Case collection

Zhongshan City People’s Hospital is a comprehensive medical centre with 81 adult intensive care beds. The clinical data for AFM patients (> 18 years old) who underwent ECMO for a poor response to drug therapy and/or intra-aortic balloon pump (IABP) therapy from January 2003 to January 2022 were reviewed. The research protocol was approved by Ethics Committee on Clinical Scientific Research and Laboratory Animal of Zhongshan People’s Hospital, and the requirement for informed patient consent was waived in view of the retrospective nature of the study.

#### 2.1.2. Case grouping

According to different treatment regimens, the AFM patients were divided into an old regimen group and a new regimen group. In the old regimen group, the ECMO treatment regimen used from January 2003 to December 2012 was adopted: for patients with Vasoactive Inotropic Score (VIS)(13) ≥ 30 and antiarrhythmic therapy were unsatisfactory, IABP was preferred. If VIS ≥ 50, SBP < 90 mmHg, blood lactic acid > 10 mmol/L, or CA, ECMO was given. In the new regimen group, the ECMO treatment strategy used from January 2013 to January 2022 was adopted: for patients with VIS ≥ 30 and antiarrhythmic therapy were unsatisfactory, SBP < 90 mmHg, blood lactic acid > 5 mmol/L, or CA, ECMO was given.

### 2.2. Methods

#### 2.2.1. Indications and timing for ECMO

ECMO was performed when one of the following three conditions occurred in AFM patients: (1) Refractory cardiogenic shock: When ≥ 2 vasoactive drugs were used, the VIS was ≥ 30, or IABP was given, radial artery systolic blood pressure (SBP) < 90 mmHg, left ventricular ejection fraction (LVEF) derived from transthoracic echocardiography < 35%, hypourocrinia, metabolic acidosis, and multiple organ dysfunction occurred. (2) Malignant arrhythmia: Third-degree atrioventricular block, ventricular tachycardia, and malignant arrhythmia failed to be corrected after treatment with antiarrhythmic drugs, electrical cardioversion/defibrillation, and temporary endocardial pacing, which was accompanied by severe hemodynamic instability. (3) CA: CA occurred during the treatment of cardiogenic shock and arrhythmia, and 5-10 min of cardiopulmonary resuscitation (CPR) could not achieve the return of spontaneous circulation (ROSC), or CA recurred after the ROSC.

#### 2.2.2. ECMO cannulation

All enrolled AFM patients underwent ECMO (Medtronic, USA) for cardiac function support. Specifically, the patients were catheterised by vascular surgeons through percutaneous femoral arterial/venous puncture or incision under anaesthesia. The distal perfusion catheter was placed prophylactically in patients catheterised through an incision. The tip of the ECMO venous outflow catheter (19-21 French) was placed in the right atrium, and the tip of the ECMO arterial inflow catheter (17-19 French) was placed in the femoral artery.

#### 2.2.3. ECMO management strategy

Individualised ECMO flow was set based on the principle of “using the lowest ECMO flow to meet the oxygen metabolism needs of the body”. The initial ECMO flow was 2.0-3.5 L/min, low-dose vasoactive drugs (VIS ≤ 15) were used to maintain a mean arterial pressure (MAP) ≥ 60 mmHg, central venous pressure (CVP) ≤ 10 mmHg, and central venous oxygen saturation (ScVO_2_) ≥ 60% and to decrease lactic acid (Lac) (with a target of < 3.0 mmol/L) at the radial artery. If the SBP, pulse-pressure difference at the radial artery, and LVEF derived from transthoracic echocardiography all increased during ECMO, the ECMO flow should be reduced by 10-20% daily. Unfractionated heparin was used for anticoagulation, with a target activated clotting time (ACT) of 160-200 s, an activated partial thromboplastin time (APTT) of 50-80 s, platelets (PLT) > 50 × 10^9^, and fibrinogen (Fib) > 1.5 g/L. The blood supply of the lower limb (skin temperature and dorsalis pedis artery pulse) on the side of femoral artery catheterization was monitored every 4 h. If the differential pulse pressure at the radial artery was < 10 mmHg and manifestations of left ventricular dilation such as pulmonary oedema emerged during ECMO, left ventricular decompression was performed by reducing the ECMO flow, increasing the dose of positive inotropic drugs, establishing a negative volume balance, and applying positive end-expiratory pressure (PEEP). Interatrial septal perforation and IABP therapy were not routinely used for left ventricular decompression.

#### 2.2.4. Drug therapy for the primary disease in AFM

All enrolled patients were routinely treated with glucocorticoids, such as dexamethasone (10 mg, IV, Bid) or methylprednisolone (40 mg, ivgtt, bid), for 3-5 day. The patients suspected of having virus-induced AFM were routinely given antiviral drugs, such as acyclovir injection (5 mg/kg, ivgtt, bid) or ganciclovir injection (5 mg/kg, iv, bid), for 5-7 d. Before 2012, IVIG was not routinely used for immunomodulatory therapy. After 2013, IVIG was routinely administered at 200-400 mg/kg/d for 3-5 days.

#### 2.2.5. Functional support of vital organs during ECMO

##### 2.2.5.1. Respiratory support during ECMO

Awake ECMO was performed if the patient had no clinical manifestations of pulmonary oedema such as shortness of breath, hypoxia, and pink frothy sputum before and after ECMO and was conscious and able to cooperate with the treatment. Invasive mechanical ventilation was used under the “lung protective ventilation strategy” if the patient had clinical manifestations of pulmonary oedema before ECMO or during awake ECMO. If pulmonary oedema was relieved but not to the degree that would weaning from ECMO, awake ECMO was used.

##### 2.2.5.2. Renal replacement therapy during ECMO

Continuous renal replacement therapy (CRRT) was performed if cardiogenic shock in AFM patients could not be corrected, manifestations of acute kidney injury such as oliguria, anuria, metabolic acidosis, and high-level creatinine emerged, the urine output did not recover (< 0.5 mL/kg/h) more than 3 h after ECMO, and diuretic drug therapy failed. Withdrawal of CRRT after improvement of acute kidney injury.

#### 2.2.6. ECMO weaning strategy

An ECMO weaning test was initiated if circulatory function improved, ECMO flow ≤ 2.0 L/min, VIS ≤ 15, SBP ≥ 90 mmHg, pulse-pressure difference ≥ 20-30 mmHg, CVP ≤ 5 mmHg, Lac ≤ 2.0 mmol/L, and LVEF ≥ 35% during ECMO. In the test, the ECMO flow was gradually reduced to 0.5-1.0 L/min, and the results were observed for more than 0.5 h. The patients were finally weaned from ECMO if VIS ≤ 15, SBP ≥ 90 mmHg, pulse-pressure difference ≥ 20-30 mmHg, CVP ≤ 5 mmHg, and LVEF ≥ 35%, without shortness of breath and hypoxia.

#### 2.2.7. Endpoints

The primary endpoint was survival to discharge, and the secondary endpoints were the recovery of cardiac function and successful weaning from ECMO.

### 2.3. Data collection

The general data for AFM patients, including gender, age, Body Surface Area (BSA), underlying diseases, and Acute Physiology and Chronic Health Evaluation II (APACHE II) score at admission to the ICU, were collected. In addition, the data before ECMO (reasons for ECMO, VIS, LVEF, MAP, Lac, myocardial injury markers, serum creatinine, etc.) and after ECMO (duration of ECMO, ECMO-related complications, the incidence of left ventricular dilation, weaning success rate, survival rate, cause of death, etc.) were also obtained.

### 2.4. Statistical analysis

Continuous variables are described as the mean ± standard deviation, and univariate comparisons between the two groups were performed using *t*-tests. Categorical variables are described as numbers, and univariate comparisons between the two groups were performed using the chi-square tests to identify the influencing factors for the survival rate. Then, multivariate logistic regression analysis was performed to identify the independent influencing factors for the survival rate. All statistical analyses were performed using SPSS 22.0 (IBM Corp., Armonk, NY, USA), and *P*-values < 0.05 were considered statistically significant.

## 3. Results

### 3.1. Demographic data and prognosis of the AFM patients

**FIGURE 1:**
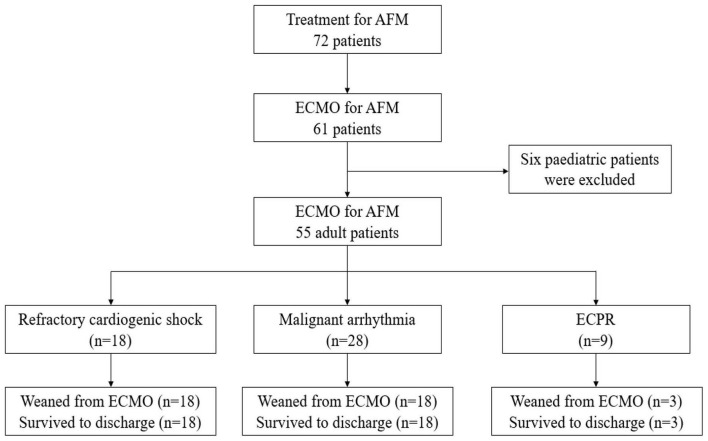
A total of 785 patients with acute myocarditis were treated from January 2003 to January 2022, including 72 AFM. Among them, 61 patients underwent ECMO, and six pediatric patients were excluded. Fifty-five patients were finally enrolled. Eighteen patients received ECMO due to refractory cardiogenic shock, 8 were successfully weaned from ECMO, and 8 were discharged. Twenty-eight patients received ECMO due to malignant arrhythmia, 28 were successfully weaned from ECMO, and 28 were discharged. Nine patients received ECMO due to malignant arrhythmia, 3 were successfully weaned from ECMO, and 3 were discharged. Outcomes and reasons of patients supported with ECMO for AFM. AFM, acute fulminant myocarditis; ECMO, extracorporeal membrane oxygenation; ECPR, extracorporeal cardiopulmonary resuscitation.

A total of 785 patients with acute myocarditis were treated from January 2003 to January 2022, including 72 AFM. Among them, 61 patients underwent ECMO, and six paediatric patients were excluded. Fifty-five patients aged (31.2 ± 11.3) years were finally enrolled, including 24 males. Forty-nine patients were weaned successfully from ECMO [duration: (4.1 ± 1.8) d], all of whom were discharged from the hospital, with a survival rate of 89.1% (Figure 1). Five patients in the old regimen group died of cardiogenic shock due to poor cardiac function during ECMO, while one patient in the new regimen group died of septic shock due to multi-resistant bacterial infections and poor cardiac function during ECMO. The follow-up results showed that all discharged patients were alive with NYHA class I cardiac function, and no case progressed to cardiomyopathy. All patients had prodromal symptoms such as fever, cough, and vomiting, 13 of whom had ECG ST-T changes at admission and tested negative on coronary angiography. No statistically significant differences in gender, age, APACHE II score on admission to ICU and other baseline data were found between the two groups (*p* > 0.05) (Table 1).

**TABLE 1:** Demographic characteristics of the new and old regimen group of AFM supported with ECMO.

Variable	Total (*n* = 55)	Old regimen group (*n* = 15)	New regimen group (*n* = 40)	*P*
Age (yrs)	31.2 ± 11.3	27.3 ± 9.1	32.7 ± 11.8	0.29
Gender				0.78
Male n (%)	24 (43.6)	7 (46.7)	17 (42.5)	
Female n (%)	31 (56.4)	8 (53.3)	23 (57.5)	
BSA (kg/m^2^)	1.3 ± 0.3	1.3 ± 0.2	1.32 ± 0.3	0.81
**Past medical history**				
Ventricular septal defect n (%)	3 (5.5)	2 (13.3)	1 (2.5)	0.12
Hypertension n (%)	1 (1.8)	0 (0)	1 (2.5)	0.54
Diabetes mellitus n (%)	1 (1.8)	0 (0)	1 (2.5)	0.54
APACHE II score on admission to ICU	28.7 ± 13.8	29.5 ± 12.1	28.3 ± 11.2	0.91

AFM, acute fulminant myocarditis; ECMO, extracorporeal membrane oxygenation; BSA, body surface area; APACHE II, acute physiology and chronic health evaluation; ICU, intensive care unit; *P < 0.05.

### 3.2. Comparison of major interventions for AFM patients between the old regimen group and the new regimen group

Compared with the old regimen group, the new regimen group had a higher proportion of IVIG treatment for AFM. Indicators reflecting the early timing of ECMO intervention included lower proportion of IABP implantation before ECMO, shorter time from onset of shock to ECMO, and lower VIS before ECMO, and the differences were statistically significant (P < 0.05). Compared with the old regimen group, the new regimen group used a distal perfusion catheterization, the proportion of temporary cardiac pacemaker and CRRT before ECMO was lower, and the flow rate after 24 h and 48 hours of ECMO were lower, and the differences were statistically significant (*P* < 0.05) (Table 2).

**TABLE 2:** Comparison of major interventions for AFM patients between the old and new regimen group.

Variable	Total (*N* = 55)	Old regimen group (*N* = 15)	New regimen group (*N* = 40)	*P*
**Drug therapy for AFM**				
Antiviral therapy *n* (%)	53 (96.3)	14 (93.3)	39 (97.5)	0.46
Glucocorticoids *n* (%)	45 (81.8)	15 (100)	38 (95)	0.38
IVIG *n* (%)	34 (61.8)	6 (40)	28 (70)	0.04*
**Early ECMO intervention**				
The duration of shock to ECMO (hr)	8.5 ± 9.7	14.1 ± 15.3	6.5 ± 5.5	<0.01*
IABP was inserted before ECMO *n* (%)	10 (18.2)	6 (40)	2 (5)	<0.01*
VIS before ECMO (μg/kg/min)	53.4 ± 29.9	87.8 ± 37.2	43.2 ± 13.9	<0.01*
**Pre-ECMO treatment**				
EC/ED *n* (%)	19 (34.5)	5 (33.3)	15 (37.5)	0.80
Temporary intracardiac pacemaker *n* (%)	8 (14.5)	4 (26.7)	4 (10)	0.01*
Initiate CRRT therapy *n* (%)	14 (25.5)	7 (46.7)	7 (17.5)	0.03*
Distal perfusion catheterization *n* (%)	48 (87.3)	8 (53.3)	40 (100)	<0.01*
**Post ECMO flow**				
ECMO flow at 24 h (L/min)	2.8 ± 0.4	3.2 ± 0.5	2.5 ± 0.3	0.04*
ECMO flow at 48 h (L/min)	2.7 ± 0.3	3.1 ± 0.2	2.4 ± 0.1	0.04*

AFM, acute fulminant myocarditis; ECMO, extracorporeal membrane oxygenation; IVIG, intravenous immunoglobulin; IABP, intra-aortic balloon pumping; VIS, vasoactive inotropic score; EC, electric compounding; ED, electrical defibrillation; CRRT, continuous renal replacement therapy; *P < 0.05.

### 3.3. Comparison of related factors before ECMO between the old regimen group and the new regimen group with early ECMO intervention

Compared with the old regimen group, the new regimen group **a**fter early ECMO intervention had a lower proportion of CA and ECPR before ECMO, and a lower proportion of ECPR among ECMO causes, the difference was statistically significant (*P* < 0.05). Compared with the old regimen, the MAP and LVEF values in the new regimen group were higher before ECMO, and the difference was statistically significant (*P* < 0.05). Compared with the old regimen, the levels of CK-MB, hs-TnT and Lac before ECMO in the new regimen group were lower, with statistical significance (*P* < 0.05) (Table 3).

**TABLE 3:** Comparison of pre-ECMO factors between the old and new regimen group after early ECMO intervention.

Variable	Total (*n* = 55)	Old regimen group (*n* = 15)	New regimen group (*n* = 40)	*P*
**Indication for ECMO**				
Cardiogenic shock *n* (%)	18 (32.7)	0 (0)	18 (45)	<0.01*
Arrhythmia (VT/VF/AVB) *n* (%)	28 (50.9)	9 (60)	19 (47.5)	0.41
ECPR n (%)	9 (16.4)	6 (40)	3 (7.5)	<0.01*
Pre-ECMO cardiac arrest *n* (%)	14 (25.5)	7 (46.7)	7 (17.5)	0.03*
**Pre-ECMO hemodynamic state**				
MAP (mmhg)	45.2 ± 22.4	30.7 ± 27.4	50.6 ± 17.8	<0.01*
LVEF	0.24 ± 0.13	0.16 ± 0.14	0.27 ± 0.11	0.02*
**Laboratory examination**				
CK (U/L)	2,800 ± 1510	4,453 ± 2,180	2,180 ± 725	0.06
CK-MB (U/L)	190 ± 164	320 ± 204	142 ± 115	<0.01*
hs-TnT (ng/l)	3,892 ± 2521	5,006 ± 2658	3,475 ± 2369	0.04*
NT-proBNP (pg/l)	17,911 ± 10,763	21,655 ± 10,153	16,507 ± 10,770	0.12
Lac (mmol/l)	8.2 ± 6.5	12.1 ± 7.1	6.7 ± 5.6	<0.01*
Cr (mmol/l)	123.5 ± 84.3	131.3 ± 127.3	120.6 ± 63.9	0.7

ECMO, extracorporeal membrane oxygenation; VT, ventricular tachycardia; VF, ventricular fibrillation; AVB, atrioventricular block; ECPR, extracorporeal cardiopulmonary resuscitation; MAP, mean arterial pressure; LVEF, left ventricular ejection fraction; CK, creatine kinase; CK-MB, creatine kinase muscle/brain; hs-TnT, human hypersensitive troponin; NT-proBNP, N-terminal pro-B type natriuretic peptide; Lac: lactic acid; Cr: creatinine; *P < 0.05.

### 3.4. Comparison of related factors after ECMO between the old regimen group and the new regimen group with early ECMO intervention, distal perfusion catheterization, treatment with IVIG and low ECMO flow

Compared with the old regimen group, the proportion of patients with coma after 48 h of CPR in the new regimen group decreased significantly after early ECMO intervention (*P* < 0.05). The incidence of severe ischemic injury of lower limbs was significantly decreased after the distal perfusion catheter was placed in the new regimen group, and the difference was statistically significant (*P* < 0.05). After ECMO with low ECMO flow and treatment with IVIG, the proportion of left ventricular dilatation decreased significantly, and the proportion of left ventricular dilatation improvement increased significantly (*P* < 0.05). Patients in the new regimen group who were successfully weaned from ECMO had shorter ECMO time and higher success rates of weaning and treatment (*P* < 0.05). According to the 1-year follow-up of discharged patients, the LVEF did not significantly differ between the two groups (*p* > 0.05) (Table 4).

**TABLE 4:** Comparison of complications and therapeutic efficacy between the old and new regimen group with optimising the ECMO treatment regimen.

Variable	Total (*N* = 55)	Old regimen group (*N* = 15)	New regimen group (*N* = 40)	*P*
**Complications of ECMO intubation**				
Ischemic leg n (%)	6 (10.9)	4 (26.6)	2 (5)	0.02*
Bleeding in the intubation area n (%)	8 (14.5)	4 (26.6)	4 (10)	0.12
Infection in the intubation area n (%)	2 (3.6)	0 (0)	2 (5)	0.38
Left ventricular dilatation n (%)	20 (36.3)	9 (60)	11 (27.5)	0.02*
Left ventricular dilatation improved n (%)	14 (25.4)	4 (44.4)	11 (100)	0.02*
Awake ECMO n (%)	12 (21.8)	3 (20)	9 (22.5)	0.08
The proportion of coma in CA after 48 h of CPR n (%)	11 (20)	7 (100)	4 (57.1)	0.02*
Weaned from ECMO *n* (%)	49 (89.1)	10 (66.7)	39 (97.5)	<0.01*
**Outcome of weaned from ECMO**				
The duration of ECMO (d)	4.1 ± 1.8	3.2 ± 1.4	4.4 ± 1.8	0.06
The duration of ICU (d)	9.1 ± 6.6	7.7 ± 2.9	9.3 ± 7.2	0.49
The duration of hospital (d)	22.1 ± 12.4	18.9 ± 9.9	22.9 ± 12.9	0.37
Survived to discharge *n* (%)	49 (89.1)	10 (66.7)	39 (97.5)	<0.01*
**Follow-up of patients 1 year after discharge**				
Hypoxic encephalopathy *n* (%)	2 (3.6)	0 (0)	2 (5)	0.18
LVEF	0.57 ± 0.27	0.58 ± 0.28	0.57 ± 0.27	0.71
Survival rate (%)	89.10	66.67	97.50	<0.01*

ECMO, extracorporeal membrane oxygenation; IVIG, intravenous immunoglobulin; CA, cardiac arrest; CPR, cardiopulmonary resuscitation; ICU, intensive care unit; LVEF, left ventricular ejection fraction. *P < 0.05.

### 3.4. Multivariate analysis: Logistic regression analysis of predictors of influencing survival rate for patients requiring ECMO for AFM

According to multivariate logistic regression analysis, the duration of shock to ECMO and the VIS before ECMO were independent risk factors for the survival rate (*p* < 0.05) (Table 5).

**TABLE 5:** Multivariate analysis: Logistic regression analysis of predictors of influencing survival rate for patients requiring ECMO for AFM.

Variable	OR (95% CI)	*P*
VIS before ECMO	1.16 (1.02-1.32)	0.02*
The duration of shock to ECMO (hr)	0.94 (0.68-1.31)	0.04*
Distal perfusion catheterization	0.85 (0.59-1.21)	0.99
Treatment with IVIG	2.99(0.02-46.71)	0.67
Left ventricular dilatation after ECMO	0.29 (0.01-8.07)	0.47

ECMO, extracorporeal membrane oxygenation; AFM, acute fulminant myocarditis; VIS, vasoactive inotropic score; IVIG, intravenous immunoglobulin; *P < 0.05.

## 4. Discussion

AFM is mainly characterised by rapid deterioration of cardiac function, and the risk of cardiac arrest is still high despite early mechanical assistance from an IABP (4, 8). Due to difficulty in ensuring the quality of CPR and in ECMO catheterization under chest compression, patients resuscitated from CA prior to ECMO are prone to severe cardiac and cerebral injury, thus affecting their prognosis (14–16). Studies have demonstrated that CA and ECPR before ECMO are independent risk factors for the prognosis of AFM patients (10, 11). In our centre, seven of 15 patients in the old regimen group suffered CA before ECMO due to late ECMO intervention, five of whom died of refractory cardiogenic shock, with a mortality rate as high as 71.4% (5/7). Therefore, reducing CA and ECPR before ECMO is the key to a higher survival rate for AFM. After January 2013, ECMO intervention was applied in advance. As a result, only seven of 40 patients had CA before ECMO, and one of three ECPR patients died, with a mortality rate as low as 14.3% (1/7). Therefore, earlier ECMO intervention is helpful for reducing CA and ECPR before ECMO in AFM patients, thereby improving the survival rate for AFM patients.

ECMO will increase left ventricular afterload. If patients have poor cardiac function before ECMO, they are susceptible to left ventricular dilatation, pulmonary oedema, and even fatal left ventricular thrombosis after ECMO (17, 18). Left ventricular dilatation following ECMO has been found to be an important risk factor for the prognosis (17–19). In the case of left ventricular dilatation following ECMO, indirect left ventricular decompression methods are usually adopted first, such as IABP, reduction in the ECMO flow and volume load, and increased doses of inotropic drugs. If the effect of indirect decompression is unsatisfactory, direct decompression methods are recommended, such as an Impella device, a left atrial cannula, or a left ventricular Apex (20). Worse cardiac functional recovery and no ECMO weaning after 3-7 days of ECMO indicate poor myocardial reversibility; therefore, ventricular assist device or heart transplantation should be considered (21, 22).

In a study conducted by the research group of Professor Chen, 23 of 75 AFM patients undergoing ECMO received direct decompression due to left ventricular dilatation; the mortality rate was as high as 52.2% (12/23) even though nine patients received left ventricular assistance device and heart transplantation after the operation (4). In our centre, left ventricular dilatation and pulmonary oedema occurred in nine of 15 patients undergoing ECMO in the old regimen group, which were all treated with indirect decompression. As a result, five patients died from a lack of improvement in left ventricular dilatation. Therefore, effective prevention and treatment of left ventricular dilatation following ECMO is the key to improving the survival rate for AFM.

After January 2013, ECMO intervention was applied in advance, and a low ECMO flow meeting the patients’ metabolic needs was adopted. As a result, the incidence of left ventricular dilatation declined from 60% to 27.5%. Moreover, indirect decompression without IABP was conducted, and the improvement rate for left ventricular dilatation increased from 44.4% to 100%, with no left ventricular thrombosis observed. The duration of ECMO was significantly shortened in patients who were successfully weaned. Earlier ECMO intervention alleviates myocardial injury, and low ECMO flow support reduces left ventricular afterload, which can help inhibit left ventricular dilatation and enhance the rapid recovery of cardiac function, thereby increasing the survival rate for AFM. Furthermore, the results of the multivariate logistic regression analysis revealed that the duration of shock to ECMO and the VIS before ECMO were independent risk factors for the survival rate. The research group of Hyeok-Hee Lee has also recently found that the timing of ECMO intervention is closely related to the prognosis of patients with refractory cardiogenic shock (23).

Viral infection is the most important cause of AFM (24, 25). Viral invasion, replication, and direct damage to the myocardium usually occur in the early stage of the disease, necessitating early antiviral therapy (26). In addition, immunity or autoimmunity is also implicated in the occurrence and development of myocardial inflammation (27). Corticosteroids plus IVIG has been found to relieve the myocardial inflammatory response (28, 29), and IVIG can downregulate proinflammatory cytokines with negative inotropic effects (30). Recently, the ability of IVIG plus corticosteroids to promote cardiac functional recovery in AFM patients has been reported (31, 32). Before 2012, patients suspected of having virus-induced AFM were primarily treated with antiviral therapy plus corticosteroids, and based on the outcomes, IVIG was generally applied after 2013, which exhibited good efficacy. Therefore, IVIG in combination with ECMO support may help ameliorate the prognosis of AFM.

## 6. Study limitation

(1) In this single-centre retrospective study, the causality between study factors and conclusions was less accurate than that in prospective randomised controlled studies. (2) Endomyocardial biopsy was not performed on patients due to the risk of worsening myocardial injury, resulting in insufficient accuracy of AFM diagnosis. (3) Since virus isolation or gene testing in relation to AFM could not be performed, the pathogen responsible for AFM was unclear. (4) There were a larger number of patients in the second group so may have better outcomes in part due to learning and experience with pts that is not otherwise measured. (5) Earlier ECMO intervention may lead to overtreatment of adult patients with AFM, but can markedly improve the treatment success rate without causing ECMO-related complications. Therefore, early ECMO use is still recommended for patients who do not respond well to conventional therapy.

## 7. Conclusion

In our experience, the duration of shock to ECMO and the VIS before ECMO are independent risk factors for the treatment success rate for adult AFM patients. Therefore, early ECMO application in adult AFM patients with a poor response to conventional therapy and low-flow support to meet metabolic needs after ECMO can not only reduce serious complications affecting the prognosis, such as CA, ECPR and left ventricular dilatation after ECMO, but also shorten the ECMO duration and facilitate cardiac functional recovery, may be associated with better outcomes.

## Data availability statement

The raw data supporting the conclusions of this article will be made available by the authors, without undue reservation.

## Ethics statement

The studies involving human participants were reviewed and approved by Ethics Committee on Clinical Scientific Research and Laboratory Animal of Zhongshan People’s Hospital. Written informed consent from the patients/participants or patients/participants next of kin was not required to participate in this study in accordance with the national legislation and the institutional requirements.

## Author contributions

LH and HL conceived the study and are responsible for the integrity of the work as a whole. LH and SZ performed the retrospective data collection and statistical analysis. LH, HL, and SZ analyzed the study data. LH wrote the initial draft of the article, which was revised by all the study authors. All authors provided input on the interpretation of the data and approved by the final study manuscript.
